# Alpine shrub growth follows bimodal seasonal patterns across biomes – unexpected environmental controls

**DOI:** 10.1038/s42003-022-03741-x

**Published:** 2022-08-06

**Authors:** Svenja Dobbert, Eike Corina Albrecht, Roland Pape, Jörg Löffler

**Affiliations:** 1 Department of Geography, University of Bonn, Meckenheimer Allee 166, Bonn, D-53115 Germany; 2 Department of Natural Sciences and Environmental Health, University of South-Eastern Norway, Gullbringvegen 36, Bø, N-3800 Norway

**Keywords:** Biogeography, Plant ecology

## Abstract

Under climate change, cold-adapted alpine ecosystems are turning into hotspots of warming. However, the complexity of driving forces of growth, associated biomass gain and carbon storage of alpine shrubs is poorly understood. We monitored alpine growth mechanisms of six common shrub species across contrasting biomes, Mediterranean and tundra, using 257 dendrometers, recording stem diameter variability at high temporal resolution. Linking shrub growth to on-site environmental conditions, we modelled intra-annual growth patterns based on distributed lag non-linear models implemented with generalized additive models. We found pronounced bimodal growth patterns across biomes, and counterintuitively, within the cold-adapted biome, moisture, and within the drought-adapted biome, temperature was crucial, with unexpected consequences. In a warmer world, the Mediterranean alpine might experience strong vegetation shifts, biomass gain and greening, while the alpine tundra might see less changes in vegetation patterns, minor modifications of biomass stocks and rather browning.

## Abstract (web summary)

Generalized additive models reveal an unexpected environmental control in shrub growth across biomes.

## Introduction

Rapid climate change is expected to impact woody plants across biomes, yet the trends observed in recent decades are seasonally heterogeneous and spatially variable^1^. An increase in temperature, outpacing the global average, is turning high-elevation ecosystems into hotspots of climate warming^2,3^. Within these emerging hotspots, life has evolutionally adapted to the physical peculiarities of the alpine environment, with temperatures being too low to support tree growth and plant species reaching their low-temperature climate limit^4^. At the same time, observed and predicted changes in this vegetation structure are expected to have a profound impact on the global carbon cycle and climate^4,5^. However, given the complexity and spatial heterogeneity of the observed trends, overarching patterns and underlying physiological processes remain poorly understood^6,7^.

In the alpine regions of the tundra biome, an intense warming trend^3,8^ has strongly enhanced shrub growth and promoted shrub encroachment at the uppermost distribution limit, leading to a wide-spread increase in biomass and coverage of dwarf shrubs^9,10^, with potential implications for uptake and storage of atmospheric CO_2_, as well as landscape greenness and associated feedbacks^3,6,7,11^. Aside from the latitudinal temperature decline, growth in these high-latitude ecosystems is thought to be mainly constrained by decreasing temperatures with increasing elevation^4^. This link to temperature and the conditions of the free atmosphere is thought to be less pronounced in shrubs, compared to trees, mainly due to their lower stature^12^. At the same time, recent studies emphasize the local impacts of other drivers on plant growth and distribution, including the duration and spatial distribution of snow cover^13,14^ and associated soil moisture regimes^7,15^. Potential shifts in the amount and seasonality of precipitation might therefore additionally affect growth of woody plants in these areas. In general, precipitation is expected to rise in most regions of the Arctic^16^, which may involve a complex interplay with the increasing evaporative demand associated with rising temperatures^7^.

In the Mediterranean biome, the climate is characterized by hot and dry summers and mild winters, with a pronounced rainfall maximum in winter and frequent extreme precipitation following the dry season^16,17^. Accordingly, shrub growth is strongly limited by drought during the summer months and thus growth might decline with warming growing seasons and increased aridification^2,18^. At the same time, growth within alpine regions is additionally constrained by comparatively cold winters with little protective snow cover^19,20^. Predicted winter warming could, thus, enhance shrub growth in these regions by reducing thermal constraints and, consequently, lengthening the growing season^21,22^.

Overall, we still lack a profound understanding on how climate change might affect radial growth, associated biomass gain, and carbon storage of shrubs across biomes^7,23–26^. Here, understanding plant physiology and the seasonality of cambial activity at time scales within a year, instead of across years, will yield important insights into the relevant mechanisms driving these processes^27^. Annual patterns in radial stem growth of woody plants of the Northern Hemisphere are determined by the physiological activity of meristems (considered as sink activity regarding CO_2_)^28,29^, including successive processes of cell expansion, thickening of the cell walls, lignification and programmed cell death^30^. Given the carbon supply from ambient CO_2_ is sufficient, these processes are in turn directly controlled by developmental and environmental constraints^31^. Radial growth is thus restricted to favourable seasons within the year^32,33^. At the same time, spatial variability in fine-scale environmental conditions caused by the heterogeneous topography of alpine regions, determines species-specific adaptations, leading to highly localised niche patterns and corresponding variations of growth mechanisms^34^.

**Fig. 1:**
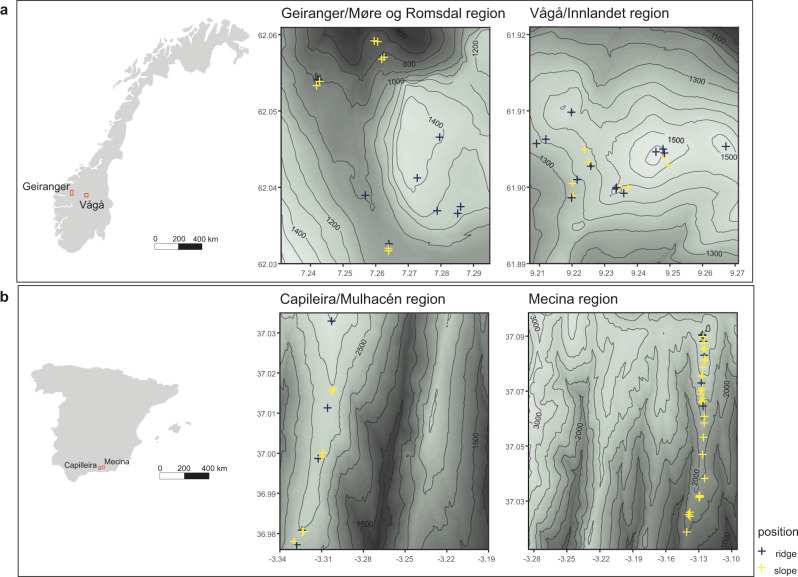
Study regions and location of study sites. The four study regions and their location in Norway (**a**) and Spain (**b**). Data source for the elevation models: Norway^75^, Spain^76^.

To monitor intra-annual and spatial patterns in radial stem expansion and contraction, xylem phenology and development, as well as their relations to the environment, high-precision dendrometers have proven to be effective tools for trees^33,35^, and, more recently, for shrubs^14,36^. Capable of recording changes at a high temporal resolution, they reveal insights at the relevant time scales of action, likely to bridge the obvious gap between plant physiology (i.e., xylem formation) and its outcome, overall radial stem growth^37^. In addition to capturing these radial growth processes the recorded raw measurements contain important information on stem swelling and shrinking resulting from water-driven turgor pressure changes in the xylem^35^. Using 257 dendrometers, we monitored growth of six common alpine shrub species, three within each the Mediterranean and the tundra biome (Fig. 1), over a consecutive period of five years. To link the observed shrub growth to measured on-site climatic conditions, we modelled intra-annual growth patterns based on distributed lag non-linear models, which were implemented with generalized additive models. Thus, we were able to focus on intra-annual dynamics of shrub growth, often overlooked in past studies, due to the low (usually annual) temporal resolution of traditional measuring methods^6,7,15^.

**Fig. 2:**
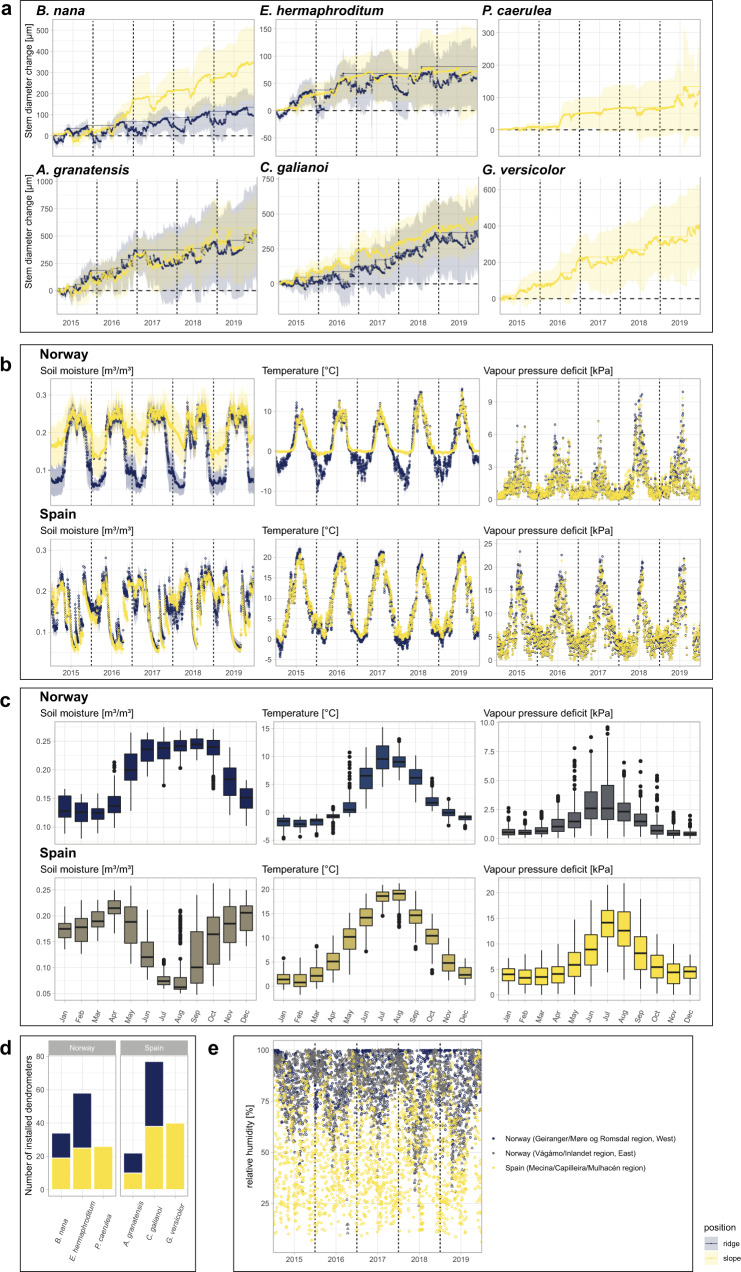
Summary of dendrometer measurements and environmental data. Averaged stem diameter change 2015–2019 (**a**) at topographical ridge (blue) and slope (yellow) positions. For *B. nana*, there was some stem increment measured during the winter of 2016 for individual specimens at the slopes, related to snow weight causing technical difficulties. Lines show cumulative stem diameter change and reversible, water deficit-induced shrinking derived from these cumulative curves. **b**, **c** Summarize daily averaged environmental data (soil moisture, soil temperature, and vapour pressure deficit) 2015–2019 (**b**) and intra-annual (monthly) variation during the same time period (**c**). The number of dendrometers installed for each species at each topographical position is presented in **d**, **e** shows relative humidity values measured at three measurement points. These values, alongside site-specific temperature measurements, were used for calculating vapour pressure deficit.

Due to the heterogeneous topography of alpine regions, both studied regions were characterized by high spatial variability in fine-scale environmental conditions, with local exposed ridges and adjoined slopes highly differentiated by snow distribution, wind, and exposure to solar radiation (Fig. 2b). These conditions, reflected in our study design, determined species-specific adaptations, leading to highly localised niche patterns and corresponding variations of growth mechanisms^34^. Still, we expected the following governing growth patterns to be reflected in our recordings:

I. For the tundra biome, xylem formation and resulting stem growth are restricted to a short phase of active xylogenesis during the warmest, snow-free parts of the year^15^, with growth processes mainly attributed to favourable thermal conditions^4,33,38^. Thus, an accelerated climate warming will most likely result in positive growth responses and, subsequently, a possible shift of species distributions to higher elevations^4,39^.
II. For the Mediterranean biome, many species show a strong bimodal pattern in annual xylem formation and stem growth, induced by summer-drought and controlled by the species’ phenology. Main growth is thus limited to phases of peak precipitation in spring and autumn^21,40–42^ and mainly controlled by water availability. Thus, warming growing seasons and increased aridification will most likely result in negative growth responses^2,18^.

## Results

**Fig. 3:**
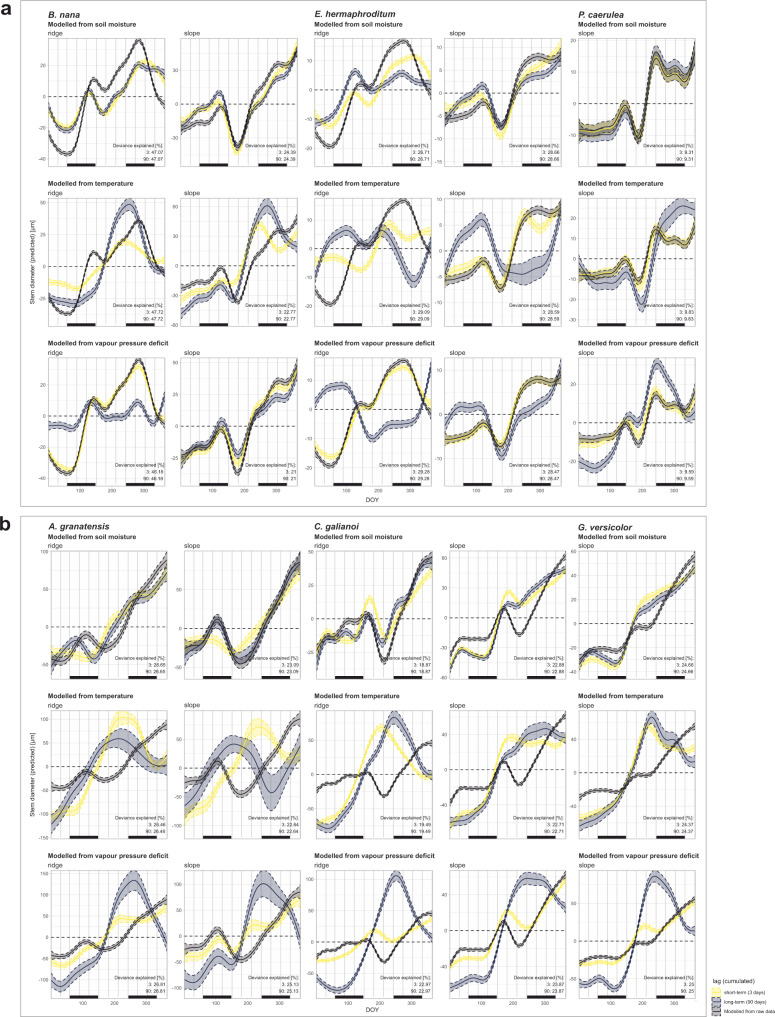
Intra-annual stem diameter change predicted by distributed lag non-linear models (DLNMs) implemented with generalized additive models (GAMs). Models for the tundra (**a**) and Mediterranean species (**b**) incorporate delayed environmental effects (soil moisture, temperature, and vapour pressure deficit) as distributed lag non-linear models (DLNMs). Colours indicate delay time spans included in the DLNMs (lag). Bars at the bottom of each graph show meteorological seasons. Coloured areas show the 95%-confidence intervals.

**Fig. 4:**
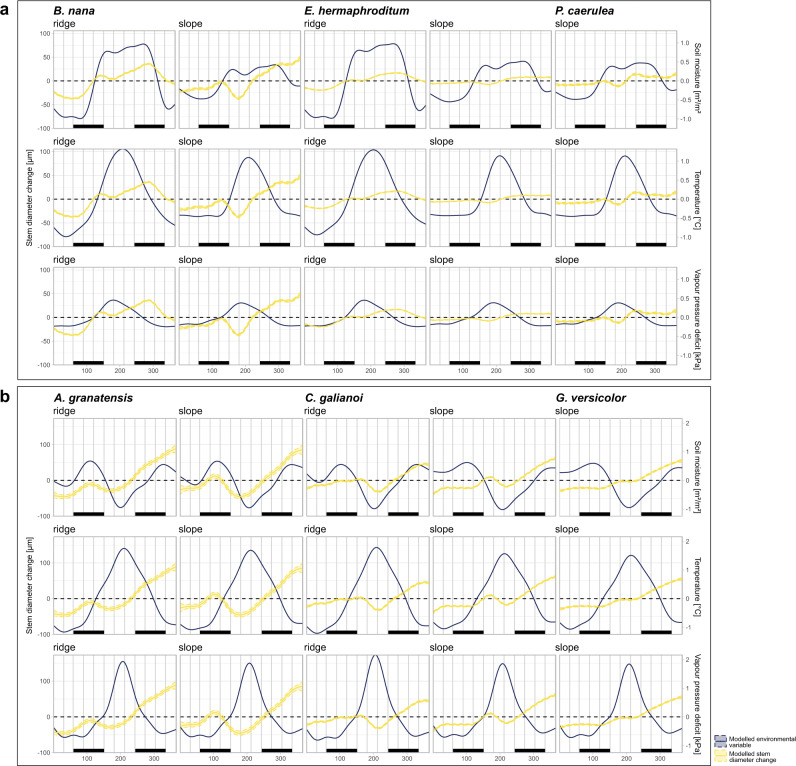
Modelled intra-annual stem diameter change and environmental variables (soil moisture, temperature and vapour pressure deficit). Each parameter was modelled from raw hourly measurements using generalized additive models (GAMs), including sampling site and year as random effects. Bars at the bottom of each graph show meteorological seasons. The tundra species are presented in **a** while **b** shows the Mediterranean species.

Mediterranean and tundra biomes are characterized by strongly differing environmental regimes (Fig. 2b, c), and our results regarding the climatic drivers of stem change suggest clear differences in climate-growth relations and, consequently, in the underlying mechanisms driving the growth processes. Overall, we found stem diameter closely linked to local micro-climate, with all three environmental variables (soil temperature (T), soil moisture (SM) and vapour pressure deficit (VPD)) strongly influencing stem diameter changes throughout the year (Figs. 3a, b, 4a, b). Differences became obvious regarding the intra-annual timing of the environmental dependencies, with effects varying strongly throughout the year and to a lesser extent between species and topographical positions.

### Do higher temperatures boost shrub growth within the alpine tundra?

**Fig. 5:**
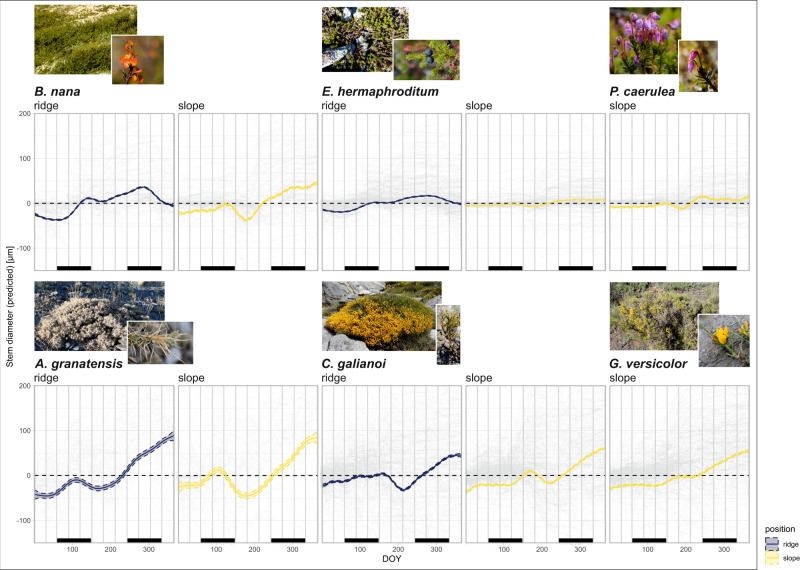
Intra-annual stem diameter change predicted by generalized additive models (GAMs). Grey dotted curves show daily means of measured stem diameter change in relation to the start of the year for all individual dendrometers. Coloured lines show modelled stem diameter change from these raw data for the six focal species and topographical positions. Sampling site and year were included as random effects into the model. Bars at the bottom of each graph show meteorological seasons. Coloured areas show the 95%-confidence intervals. Photos show one representative specimen of each species and were taken by the authors.

Our focal species in the tundra biome usually started radial stem expansion in spring (March–April, Fig. 5), in close accordance with the rise in spring soil moisture, but before the gradual rise of soil temperatures, possibly caused by delayed soil warming after snow melt (Fig. 3a). This initial stem increment early in the year was complimented by a much more pronounced growing phase in late summer (Fig. 5). For the tundra species this main phase of summer growth was promoted by short- and long-term thermal conditions and ceased with the sharp decline in soil moisture associated with freezing soils or snow, regardless of topographical position (Figs. 3a, 5). Moreover, at the exposed ridge positions of the tundra biome, where protective snow cover is usually missing and winter temperatures consequently drop significantly (Fig. 2b), we found summer growth cessation followed by a phase of radial stem shrinking during the winter months (Fig. 5). In addition, growth patterns of the tundra shrub species were closely linked to short-term fluctuations in VPD (Fig. 3a).

Overall, our results reveal growth in the alpine tundra environment to be strongly synchronised in timing and highly seasonal, with radial stem increment mainly limited to the snow-free period in spring and summer (Fig. 5) and highly adapted to seasonal temperature fluctuations and annual cycles of soil freezing and thawing (Fig. 3a). However, in addition to the expected growth limitation during the winter months, associated with low temperatures, soil freezing, and snow cover, our focal species experienced a phase of growth cessation or even active stem shrinking during summer, leading to unexpected bimodal patterns of stem diameter change (Fig. 5). This bimodality was present in both, deciduous (*Betula nana*) and evergreen (*Empetrum nigrum* ssp. *hermaphorditum* and *Phyllodoce caerulea*) species, yet varied in markedness and timing, with *E. hermaphroditum* showing the least pronounced bimodality in the species’ annual pattern of stem change (Fig. 5). Moreover, this phase of growth cessation during early summer seemed to be related to high temperatures, as well as rising vapour pressure deficit and lowering soil moisture (Figs. 4a, 5), indicating a link to increased transpiration and water depletion after the first rise of soil moisture in spring. Thus, although warming conditions initially promoted growth within arctic-alpine ecosystems, extreme summer temperatures and associated moisture limitation inhibited summer growth, thereby limiting growth processes during the snow-free period.

### Does drought hinder shrub growth within the Mediterranean alpine?

Similar to the patterns observed in the tundra biome, growth in the Mediterranean biome usually started with an initial radial stem expansion in spring or early summer (Fig. 5). For the Mediterranean species *Cytisus galianoi* and *Genista versicolor* we found this early growth onset to be mainly affected by short-term rising temperatures and soil moisture across all topographical positions (Figs. 3b, 4b). In contrast, for the species *Astragalus granatensis*, the onset of growth showed an overall low synchrony with our measured environmental parameters, with stem expansion starting very early, already before the spring rise in temperatures occurred (Fig. 4b). The early phase of radial stem increment was followed by a strongly pronounced phase of summer stem shrinkage in the Mediterranean species, mainly related to short-term fluctuations in VPD, yet in many cases starting before the main summer rise in VPD (Fig. 4b). In addition, the observed growth cessation during the summer months was related to soil moisture conditions throughout the year (Figs. 3b, 4b) and resulted in a strong bimodal pattern of stem diameter change with a high annual amplitude (Fig. 5). In line with this, we additionally observed seasonal dimorphism in the Mediterranean-alpine species *A. granatensis*. Growth started again in late summer, closely linked to available soil moisture, with short- and long-term moisture availability equally important (Fig. 3b). This main growth period continued well into the meteorological winter months, leading to an overall higher stem increment compared to the tundra species, which mostly occurred later during the year (Fig. 5).

Expectedly, growth patterns of the Mediterranean species were closely linked to moisture availability throughout the year, with stem diameter changes mainly controlled by summer drought and winter precipitation (Fig. 3b). Here, we identified the overall low soil moisture availability as a main factor in limiting radial stem expansion during the summer months, while low temperatures were crucial in limiting stem expansion during winter (Fig. 3b). Thus, we found the species’ growth processes mainly limited to phases of sufficient soil moisture during spring and autumn (Fig. 4b), continuing into the winter months provided that thermal conditions were favourable.

## Discussion

For the tundra biome, it is generally assumed that radial growth of woody plants is mainly restricted to a short phase of active xylogenesis during the warmest, snow-free parts of the year from June to August^14^, with growth onset in spring triggered by gradually rising temperatures or temperature thresholds^11,33^. Here, we found an earlier onset of radial stem expansion in all three tundra species, closely linked to the rise of available soil moisture associated with thawing soils or snow melt. Sufficient soil water availability has been reported to replenish stem water, associated with raising turgor pressure thus enabling growth processes in these early flowering species^23,35,43,44^, linking growth closely to snow cover and the length of the growing season^42,45^. In accordance with past studies ^e.g.^^31^, our results did not indicate any influences of carbon depletion, limiting such growth processes at the start of the growing season, but late frost events might limit early season cambial activity in the tundra biome^46^. Here, shrubs have developed important adaptive mechanisms for survival during the extreme winter conditions characterizing the region, including a phase of active cell water reduction to avoid cell damage at the exposed ridge positions, where a protective snow cover is missing^14,17^. Accordingly, radial stem growth ceased during autumn in our species, and we found the timing of this growth cessation closely linked to the drop in soil moisture associated with soil freezing. The cessation of seasonal wood formation was thus mostly decoupled from the gradual decline of temperatures in late summer and autumn, contradicting previous studies^33^. Yet, during the preceding main phase of radial growth in late summer our results confirm a thermal control of stem growth ^e.g.^^33^, with strong additional links to soil moisture availability, especially at the slopes, as well as short-term fluctuations in atmospheric vapour pressure deficit in both evergreen and deciduous species. Thus, for the tundra species, we can confirm the overall importance of the duration of favourable conditions during the summer months (growing season) for xylogenesis and consequent stem increment during this main growth phase^38,47^. However, while growth processes in arctic-alpine environments are usually mainly attributed to temperatures^4,33,38^, our results highlight the importance of thermally controlled soil moisture availability, in this context, pointing towards an emerging warming-induced moisture limitation^48^.

Across cold climates, shrub growth has been shown to respond positively to rising temperatures, with special emphasis on early season conditions^49^, while only a small number of studies has reported a negative growth response to summer warming^36,39,50,51^. An accelerated climate warming is thus predicted to result in positive growth responses and, subsequently, a possible shift of species distributions to higher elevations^4,39,50^. Yet, our results imply a clear limitation of summer growth, suggesting that radial growth in shrubs of the winter-dry tundra biome follows a bimodal pattern, with a phase of stem diameter shrinking or growth cessation during early summer. We found the timing of this summer stem shrinkage in the tundra biome to be closely linked to summer temperature and VPD maxima, being most pronounced during exceptionally warm summers, especially for the evergreen species *E. hermaphroditum* and *P. caerulea*. The limitation of summer growth can therefore be interpreted as a reaction to unusually high summer temperatures, triggering increased transpiration after depletion of snowmelt water in spring, which brings growth processes to a halt^35,52^. Thus, the observed summer dormancy of tundra shrubs is most likely a result of the species’ sensitivity to changing thermal conditions, with rapidly warming summers^1,8^ and positive effects of the predicted lengthening of the growing season due to climate change^3^ might be counteracted by these environmental constraints.

While our findings confirmed overall positive effects of temperatures on growth rate and duration, rising summer temperatures will most likely limit cambial activity during the main growing season^47,50^, while sub-zero temperatures and soil freezing constrain growth processes during the winter months. Thus, we conclude that warming summers in the tundra biome might not, as generally assumed, promote but rather inhibit overall growth for these cold-adapted species. With their cambial rhythm strongly controlled by thermal conditions influencing soil freezing and thawing and associated soil moisture availability, woody shrubs of the tundra biome will thus most likely show negative growth responses in a warming environment.

For trees in the summer-dry Mediterranean areas a strong, drought-controlled bimodal pattern in annual xylem formation and stem growth has long been described, with growth being limited to spring and autumn^22,40–42^. In our study, we found similar bimodal patterns in radial stem variation of woody shrubs of the Mediterranean biome. Here, first radial stem increment took place in spring, usually between February and May. For two of the monitored species (*C. galianoi* and *G. versicolor*) our results confirm a thermal control on this onset of the growing season, reported in past studies across varying environments^11,33,41^. For these species, low temperatures during late winter, experienced especially at the exposed ridge positions, are presumably limiting growth, causing a short period of winter quiescence^17^, with spring precipitation playing an additional role^13^. Contrastingly, we found early season stem increment of *A. granatensis* mostly decoupled from the measured environmental controls and therefore possibly related to internal drivers associated with photoperiod^49,53,54^.

The first phase of stem growth was followed by a period of radial stem shrinkage observed in our focal species during the summer months, which can be interpreted as an interruption of growth processes, accompanied by an additional, probably water-related, shrinkage of the stem^37^. In previous studies in the Mediterranean, the cause for such summer quiescence has been described as a limitation of cambial activity to acclimatize to summer droughts, and more favourable growing conditions during spring and autumn, including mild temperatures and adequate soil water availability^17,40^. Our results confirm this assumption to some extent, linking summer stem shrinkage of the Mediterranean species mostly to the increased aridity experienced during the summer months, with rising atmospheric vapour pressure deficit and limited soil moisture, which in combination create an imbalance between transpiration and water uptake, lowering turgor pressure and impeding radial stem growth^34,43^. At the same time, our results reveal that summer shrinkage of the Mediterranean species usually starts shortly before the main summer rise in VPD and is additionally related to long-term dry conditions and limited soil water throughout the year, especially at the exposed ridges, which are also characterized by lower soil moisture availability during the winter months. Compared to the stress-related summer shrinkage observed in the tundra biome, we therefore conclude that, in contrast to trees^13^, the annual cambial rhythm of Mediterranean-alpine shrub species is highly adapted to the dry environment and endogenously controlled^41,55^, with differing levels of drought-adaptation between species. Here, the biannual leaf-forming processes observed in *A. granatensis* are a strong indicator for this adaptation.

In general, dendroecological studies of Mediterranean woody plants agree that major growth processes take place in spring, with the second phase of stem increment in autumn playing only a minor role for xylogenesis^12,13,24,41,56^. For our focal species, however, our results imply a strong role of a second growing phase. The phase of radial stem expansion continued throughout autumn and well into the winter months, lacking a clearcut growth cessation. This additional growth can be attributed to continued cell division and cell expansion, promoted by autumn and winter precipitation^17,56^. Here, winter soil temperatures had no limiting effect at the slopes, where a discontinuous snow cover prevented soil freezing. Yet, we found winter stem growth slightly affected by the lower temperatures and occasional soil freezing characterizing the snow-free ridge positions. Thus, our results support previous studies, suggesting that winter warming might promote overall radial stem growth of Mediterranean shrub species^21,22^, especially at exposed ridge positions. This implies that in a region previously perceived as primarily drought-controlled, all three focal species might become increasingly dependent on favourable winter temperatures, allowing for continued cambial activity, resulting in an overall increase in realized stem growth and associated CO_2_ uptake. At the same time, however, future changes in winter rainfall^2^ might have negative effects.

Overall, we found Mediterranean-alpine shrub species strongly adapted to severe summer-drought, with their cambial rhythm closely linked to seasonal water dynamics. This adaptive mechanism makes these species less sensitive to environmental changes of their immediate environment and might mitigate predicted negative effects of increased aridification. In a warming environment, they might thus profit from a consequent temperature-induced lengthening of the growing phase into the winter months.

In close accordance with past studies ^e.g.^^14,18^, we found radial stem changes of our focal shrub species across two strongly disparate biomes to be controlled by the micro-environmental conditions of their immediate, near- and below-ground surroundings. Water-use dynamics related to snow distribution and soil freezing, as well as atmospheric drought played a major role here. The annual patterns of radial stem diameter change revealed clear periodic annual cycles of cambial activity and dormancy, closely linked to the climatic regimes of the respective biomes. Overall, we can therefore confirm that shrubs, similar to trees^53,57^, develop highly adapted strategies of wood formation to function optimally in local conditions. Yet, such adaptation to macro- as well as micro-climatic conditions becomes critically challenged in a rapidly changing environment^54,58^, where climatic controls are shifting. With the intense warming experienced within alpine ecosystems^2,3,8^, extreme conditions are emerging as crucially growth limiting factors^4^. Thus, common preconceptions regarding secondary growth in summer-dry vs. winter-dry environments will no longer hold true. Our findings reveal that a profound understanding of growth dynamics and response mechanisms under climate change scenarios has to take into account the fine-scale, intra-annual seasonality of cambial activity and climate-growth relations. Advancing established scientific measurement methods, such as using fine-scale, hourly dendrometer measurements to model intra-annual stem diameter changes in shrub species across biomes, led to unexpected insights here: We found shrub species of both biomes capable of shifting their cambial activity towards favourable periods of the year, with a distinct phase of summer quiescence controlled by environmental constraints (rising summer temperatures in the tundra biome, summer-drought in the Mediterranean), resulting in the observed bimodal growth patterns. Enhanced shrub growth, as observed in the alpine tundra^9,10^, is therefore limited to areas of sufficient water availability during these periods. Where winter soil freezing and snow cover is crucially limiting soil moisture availability, growth processes of woody shrubs might become increasingly constrained. The Mediterranean-alpine shrub species, however, might increase in biomass where warming winters are promoting shorter periods of snow cover and a consequent lengthening of the growing phase into the winter months. Unexpectedly, in a potentially warmer world, the Mediterranean-alpine ecosystems might experience strong positive vegetation shifts, biomass gain and alpine greening, while the alpine tundra ecosystems might see less changes in vegetation patterns, minor modifications of their biomass stocks and rather alpine browning.

## Methods

### Study sites

The study was conducted in four alpine locations, two in Mediterranean-alpine climatic regimes in the Sierra Nevada, Spain, and two in arctic-alpine climate regimes in the Central Norwegian mountains (Fig. 1). Each location was situated above the local treeline and characterized by a mixture of evergreen and deciduous shrub vegetation within a topographically heterogeneous landscape. Following the framework of our long-term alpine ecosystem research project (LTAER), the monitored sites at each location were placed at ~100-m intervals from the treeline upwards and stratified-randomly chosen to cover the elevational gradient. Within each elevational band, we monitored shrub growth and micro-site environment at distinct micro-topographic positions on local, exposed ridges and adjoined slopes. In accordance with the topography, these microsites experience clearly differing environmental conditions (Fig. 2b), with the differences mainly caused by snow distribution, wind, and exposure to solar radiation. Thus, wind-blown ridges are characterised by discontinuous snow cover and deeply frozen ground in winter in the arctic-alpine areas. Due to the general absence of a thick layer of snow in winter, this effect was less visible at the Mediterranean ridges, which mainly distinguished themselves through slightly dryer conditions throughout the year.

At each site, we monitored the dominating shrub species, avoiding specimens growing near stones and small depressions, inside the radius of other larger shrub species, and near patches of wind erosion. The focal species were *Astragalus granatensis* (Lamarack), *Cytisus galianoi* (Talavera & Gibbs) and *Genista versicolor* (Boiss.) in Spain, and *Betula nana* (Linnaeus), *Empetrum nigrum* ssp. *hermaphroditum* (Hagerup) and *Phyllodoce caerulea* (Linnaeus) in Norway (Table 1). All six species were analysed for ~6 years between 01 January 2015 and 31 July 2020.

**Table 1:** Shrub species and their characteristics.

Species	Dendro-metres [*n*]	Sampled specimens [*n*]	Positions	Elevation [m a.s.l.]	Country
*Astragalus granatensis*	22	12	Ridges/Slopes	2672–2766	Spain
*Cytisus galianoi*	77	37	Ridges/Slopes	2100–3175	Spain
*Genista versicolor*	40	22	Slopes	1830–2720	Spain
*Betula nana*	34	34	Ridges/Slopes	768–1510	Norway
*Empetrum nigrum* ssp. *hermaphroditum*	58	58	Ridges/Slopes	768–1565	Norway
*Phyllodoce caerulea*	26	26	Slopes	768–1534	Norway

Dendrometers: Number of dendrometers mounted to specimens of the species (see also Fig. 1d). Sampled specimens: Number of specimens to which dendrometers were mounted. Positions: Topographical positions at which specimens were monitored. Due to species-specific environmental restrictions, two species occur on slopes only. Elevation: range of elevation at which specimens of the species were monitored.

### Experimental design

We used continuous (hourly) stem diameter data from each site, measured in parallel with on-site environmental information on air (vapour pressure deficit, VPD) and soil (temperature and moisture). To monitor stem diameter variation, we mounted high-precision dendrometers (type DRO; Ecomatik, Dachau/Germany) on above-ground stems horizontal to the ground surface for each specimen, as close to the assumed root collar as possible (~1–5 cm above the ground). If possible, we mounted dendrometers to multiple stems of the same specimen to ensure a close representation of overall radial growth. During the mounting process, we removed the dead outer bark to place the sensor as close to the living tissue as possible, following a common practice for dendrometer measurements of trees ^e.g.^^14,59^. This ensures that hygroscopic shrinkage and swelling of dead tissues from the outer bark does not influence the diameter measurements. The sensor used had a temperature coefficient of <0.2 µm/K.

### Climate and micro-environmental data

We collected continuous (hourly) data on on-site environmental conditions directly at each site, capturing the local micro-conditions at which the sampled specimens grow (Fig. 2b, c). These measurements included soil temperatures (temperature, *T*, °C) at a depth of 15 cm below the ground surface (within the root zone), which we assessed using ONSET’s type S-TMB-002 temperature sensors (±0.2 °C accuracy). Volumetric soil water content (soil moisture, SM, m³ water/m³ soil), also at 15 cm below the ground surface, was measured using ONSET’s type S-SMD-M005 soil moisture sensors (±3% accuracy). Data were measured at 1 min intervals and recorded as hourly means, using ONSET’s HOBO loggers (type 21-002). In addition, we calculated site-specific estimates of atmospheric water demand (vapour pressure deficit, VPD, kPa) based on relative air humidity (Fig. 2e) at the ridge positions, which was obtained from Skye rht+ sensors (SKH 2065) mounted to an ADL-MX datalogger (Meier NT) and our above temperature measurements at each site, using the Magnus equation of Sonntag^60^. VPD reflects the combined effect of temperatures and air humidity, and is known to directly affect plant water use through transpiration, and productivity^51^. All data, including dendrometer as well as environmental measurements are available in separate publications^61,62^.

### Statistics and reproducibility

All analyses were carried out in the R environment version 4.0.3^63^. To study and compare annual patterns and trends of stem change, we fitted generalized additive models (GAMs)^64^ to our dendrometer measurements, using the mgcv package^65^. GAMs are semi-parametric regression models that use a sum of smooth functions to model non-linear patterns in the response variable, which makes them especially useful for modelling bimodal or asymmetric data and detecting flexible dependencies^66^. Compared to parametric models, GAMs allow the data to determine the shape of the response curves and make no a priori assumption about the functional relationship between the variables^67^. Here, the response variable was hourly stem diameter change (μm) compared to the start of the study period (2014). Stem diameter change was first modelled as a smoothing function of seasonality, expressed as day of the year. Sampling site and year were included as random effects into the model to account for intra-annual and inter-site variation. The final models thus had the form

1 $${{{{{\rm{g}}}}}}({{{{{\rm{E}}}}}}({{{{{\rm{Stem}}}}}}\; {{{{{\rm{diameter}}}}}}\; {{{{{\rm{change}}}}}}_i))={{{{{\rm{a}}}}}}+{{{{{\rm{s}}}}}}1({{{{{\rm{day}}}}}}\; {{{{{\rm{of}}}}}}\; {{{{{\rm{year}}}}}}_i)+{{{{{\rm{site}}}}}}_i+{{{{{\rm{year}}}}}}_i+{{{{{\rm{\varepsilon }}}}}}_i$$

where s1 represents a smoothing function, fitted using a penalized thin plate regression spline, a is the intercept of the model, and *ε*_*i*_ is the residual value. The basis dimension (k) for the smooth was set to 12. Whether this value is adequate was checked by computing an estimate of the residual variance (based on differencing residuals that are near neighbours according to the (numeric) covariates of the smooth) divided by the residual variance (k-index)^64^. In addition, the generalized cross-validation score (GCV), as an estimate of the mean square prediction error, was used for model selection (Supplementary Fig. 1). Model diagnostics were produced using the gam.check function provided in the mgcv package^65^ and are presented in Table 2. We fitted GAMs for each species and each topographical position separately. Also, in addition to modelling stem diameter change, we fitted GAMs of a similar form to the measured environmental parameters for a direct comparison of the resulting models.

**Table 2:** Diagnostic information for the fitted generalized additive models (GAMs).

Species	Topographical position	Degrees of freedom	edf	k-index
*Astragalus granatensis*	Ridge	11	8.58	1.03
	Slope	11	9.28	1.00
*Cytisus galianoi*	Ridge	11	10.60	1.03
	Slope	11	10.70	1.10
*Genista versicolor*	Slope	11	9.43	1.02
*Betula nana*	Ridge	11	10.80	1.02
	Slope	11	10.70	1.00
*Empetrum nigrum* ssp. *hermaphroditum*	Ridge	11	10.50	1.00
	Slope	11	9.98	1.01
*Phyllodoce caerulea*	Ridge	11	10.50	1.00

Degrees of freedom: model degrees of freedom, edf: effective degrees of freedom, k-index: estimate of the residual variance divided by the residual variance^61^. This index should not be <1.

Subsequently, we included standardized environmental effects as covariates into the model structure using distributed lag non-linear models (DLNMs), implemented with the GAM^68,69^ to describe delayed effects of micro-climate on stem diameter patterns. These models allow for a detailed analysis of climate-stem diameter relations throughout the course of the year, including fine-scale seasonality of these relations. Relating models incorporating micro-climatic factors across different delayed time-frames, and models fitted to our raw stem diameter data, allow for an intuitive, visual interpretation of the results (Figs. 3, 5). Distributed lag models (DLMs) were originally proposed by Almon^70^ and are since used to describe delayed associations between an input variable and a response variable in a variety of fields [e.g., ref. ^71^]. Multiple studies have shown the importance of delayed effects of climate on vegetation growth^69,72^. DLNMs include such effects and allow for the dependence to vary smoothly over lag time by incorporating a crossbasis function, which is a combination of two functions specifying the relationships in the dimensions of predictor (predictor-response function) and lags (lag-response function), respectively^68^. For both functions, we applied a penalized and natural cubic spline function with 4 and 3 degrees of freedom, respectively, based on the Akaike information criterion^73^. The full models can thus be expressed as:

2 $${{{{{\rm{g}}}}}}({{{{{\rm{E}}}}}}({{{{{\rm{Stem}}}}}}\; {{{{{\rm{diameter}}}}}}\; {{{{{\rm{change}}}}}}_i))=	\; {{{{{\rm{a}}}}}}+{{{{{\rm{s}}}}}}1({{{{{\rm{day}}}}}}\; {{{{{\rm{of}}}}}}\; {{{{{\rm{year}}}}}}_i)+{{{{{\rm{site}}}}}}_i+{{{{{\rm{year}}}}}}_{i}\\ 	 +{{{{{\rm{s}}}}}}2({{{{{\rm{environmental}}}}}}\; {{{{{\rm{value}}}}}}_{i,t,}\,\\ 	\ldots ,{{{{{\rm{environmental}}}}}}\; {{{{{\rm{value}}}}}}_{i,t}-{{{{{\rm{lag}}}}}})+\varepsilon _i$$

where s2 is the crossbasis function, created using the dlnm package^74^. In order to meaningfully capture and differentiate delayed environmental effects on stem diameter change, we compared models for each environmental parameter (T, SM and VPD), as well as models allowing for different maximum lags, ranging from 3 to 180 days.

### Reporting summary

Further information on research design is available in the Nature Research Reporting Summary linked to this article.

## Supplementary information

Supplementary Information

Reporting Summary

## Data Availability

All underlying data pertinent to the results presented in this publication are publicly available in two separate data papers published in “ERDKUNDE---Archive for Scientific Geography” (https://www.erdkunde.uni-bonn.de). All data for the study regions in Norway is available under the 10.3112/erdkunde.2021.dp.01 and data for the study regions in Spain is available under the 10.3112/erdkunde.2022.dp.01.

## Code availability

All custom code used to generate the results presented in this study was written using the R statistical software version 4.0.3 (R Core Team, 2020). It will be made available upon request.
