# Supplementary material for: Alpine shrub growth follows bimodal seasonal patterns across biomes – unexpected environmental controls
Source: Commun Biol. 2022 Aug 6;5:793. doi: 10.1038/s42003-022-03741-x (PMC9357034; doi:10.1038/s42003-022-03741-x)
Supplement: Supplementary file 1 — Supplementary Information [file 42003_2022_3741_MOESM1_ESM.pdf]

Supplementary Information

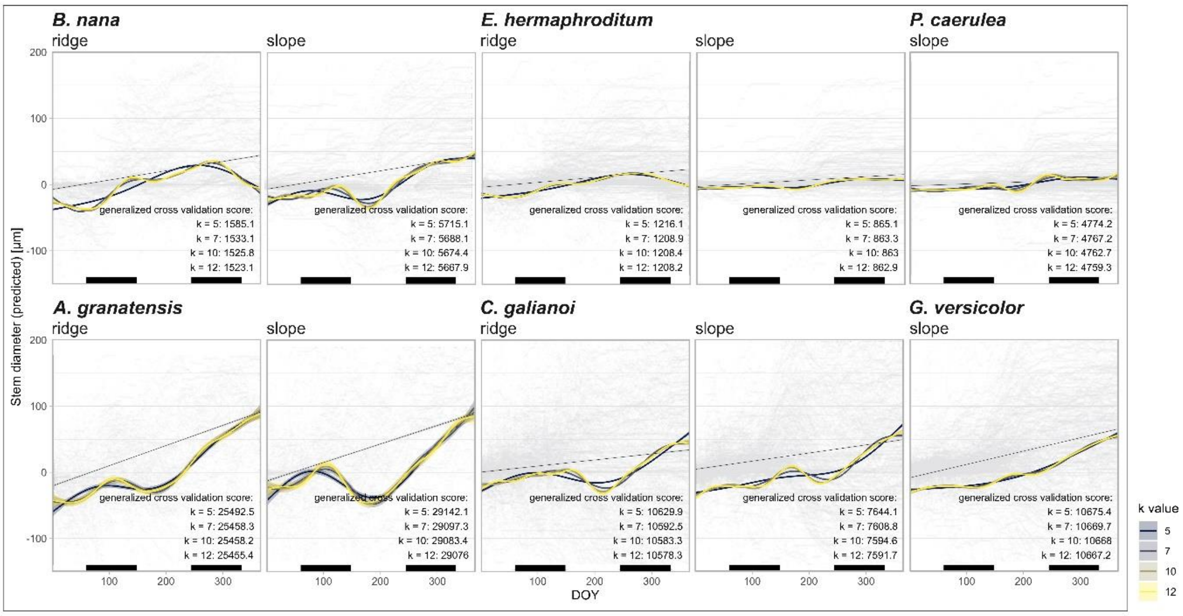

Supplementary Fig. 1: Additional generalized additive models (GAMs) predicting intra-annual stem diameter change. Grey dotted curves show daily means of measured stem diameter change in relation to the start of the year for all individual dendrometers. Colored lines show modelled stem diameter change from these raw data for the six focal species and topographical positions. Sampling site and year were included as random effects into the models. Colors indicate the k value for the respective models. Bars at the bottom of each graph show meteorological seasons. Colored areas show the 95%-confidence intervals.
