# Supplementary material for: Alpine shrub growth follows bimodal seasonal patterns across biomes – unexpected environmental controls
Source: Commun Biol. 2022 Aug 6;5:793. doi: 10.1038/s42003-022-03741-x (PMC9357034; doi:10.1038/s42003-022-03741-x)
Supplement: Supplementary file 2 — Reporting Summary [file 42003_2022_3741_MOESM2_ESM.pdf]

nature portfolio

Corresponding author(s): Dobbert, Svenja; Albrecht, Eike; Pape, Roland; Löffler, Jörg

Last updated by author(s): Jun 29, 2022

Reporting Summary

Nature Portfolio wishes to improve the reproducibility of the work that we publish. This form provides structure for consistency and transparency in reporting. For further information on Nature Portfolio policies, see our Editorial Policies and the Editorial Policy Checklist.

Statistics

For all statistical analyses, confirm that the following items are present in the figure legend, table legend, main text, or Methods section.

n/a	Confirmed	
☐	☒	The exact sample size (*n*) for each experimental group/condition, given as a discrete number and unit of measurement
☐	☒	A statement on whether measurements were taken from distinct samples or whether the same sample was measured repeatedly
☐	☒	The statistical test(s) used AND whether they are one- or two-sided<br>*Only common tests should be described solely by name; describe more complex techniques in the Methods section.*
☐	☒	A description of all covariates tested
☒	☐	A description of any assumptions or corrections, such as tests of normality and adjustment for multiple comparisons
☐	☒	A full description of the statistical parameters including central tendency (e.g. means) or other basic estimates (e.g. regression coefficient) AND variation (e.g. standard deviation) or associated estimates of uncertainty (e.g. confidence intervals)
☒	☐	For null hypothesis testing, the test statistic (e.g. *F*, *t*, *r*) with confidence intervals, effect sizes, degrees of freedom and *P* value noted<br>*Give P values as exact values whenever suitable.*
☒	☐	For Bayesian analysis, information on the choice of priors and Markov chain Monte Carlo settings
☐	☒	For hierarchical and complex designs, identification of the appropriate level for tests and full reporting of outcomes
☐	☒	Estimates of effect sizes (e.g. Cohen's *d*, Pearson's *r*), indicating how they were calculated

Our web collection on statistics for biologists contains articles on many of the points above.

Software and code

Policy information about availability of computer code

Data collection	For data collection the R Statistical software version 4.0.3 (https://www.r-project.org/) was used.
Data analysis	The data analysis presented in this study was conducted using the R Statistical software version 4.0.3 (https://www.r-project.org/). The custom code was specifically written for this project and can be made available upon request. The mathematical approach, including specific formulas, is described in detail in the Methods section of the manuscript.

For manuscripts utilizing custom algorithms or software that are central to the research but not yet described in published literature, software must be made available to editors and reviewers. We strongly encourage code deposition in a community repository (e.g. GitHub). See the Nature Portfolio guidelines for submitting code & software for further information.

Data

Policy information about availability of data

All manuscripts must include a data availability statement. This statement should provide the following information, where applicable:

- Accession codes, unique identifiers, or web links for publicly available datasets
- A description of any restrictions on data availability
- For clinical datasets or third party data, please ensure that the statement adheres to our policy

All underlying data pertinent to the results presented in this publication are publicly available in two separate data papers published in "ERDKUNDE---Archive for Scientific Geography" (https://www.erdkunde.uni-bonn.de). All data for the study regions in Norway is available under the DOI: 10.3112/erdkunde.2021.dp.01 and data for the study regions in Spain is available under the DOI: 10.3112/erdkunde.2022.dp.01.

Field-specific reporting

Please select the one below that is the best fit for your research. If you are not sure, read the appropriate sections before making your selection.

☐ Life sciences ☐ Behavioural & social sciences ☒ Ecological, evolutionary & environmental sciences

For a reference copy of the document with all sections, see nature.com/documents/nr-reporting-summary-flat.pdf

Ecological, evolutionary & environmental sciences study design

All studies must disclose on these points even when the disclosure is negative.

Study description	For this study we measured radial stem diameter change of six shrub species using 257 dendrometers (139 in the Mediterranean and 118 in the tundra biome), producing hourly data. In total 189 individual specimens were sampled, with up to five dendrometers installed to one specimen.<br>The sampled specimens were located at 101 individual sites within four study areas, with multiple species present at some sites. At each site we additionally measured near and below ground environemtal conditions (soil temperature and moisture, vapour pressure deficit).
Research sample	At each site, we monitored the dominating shrub species. The focal species were Astragalus granatensis (Lamarack), Cytisus galianoi (Talavera & Gibbs) and Genista versicolor (Boiss.) in Spain, and Betula nana (Linnaeus), Empetrum nigrum ssp. hermaphroditum (Hagerup), and Phyllodoce caerulea (Linnaeus) in Norway. The sampled specimens were chosen to reflect the environmental heterogeneity of both biomes: The two study areas in Norway represent the steep oceanic-continental gradient characterizing the region, while the individual sites were placed along the elevational gradient from the treeline upwards.
Sampling strategy	To monitor stem diameter variation, we mounted high-precision dendrometers on above-ground stems horizontal to the ground surface for each specimen, as close to the assumed root collar as possible. We avoided specimens growing near stones and small depressions, inside the radius of other larger shrub species, and near patches of wind erosion. If possible all available specimens at each site were sampled. Sites were chosen at approximately 100-m intervals from the treeline upwards to the uppermost dirspribution limit and stratified-randomly to cover the elevational gradient. Within each elevational band, we monitored shrub growth and micro-site environment at distinct micro-topographic positions on local, exposed ridges and adjoined slopes.
Data collection	To monitor stem diameter variation, we mounted high-precision dendrometers (type DRO; Ecomatik, Dachau/Germany) on above-ground stems horizontal to the ground surface for each specimen, as close to the assumed root collar as possible (approximately 1-5 cm above the ground). If possible, we mounted dendrometers to multiple stems of the same specimen to ensure a close representation of overall radial growth. During the mounting process, we removed the dead outer bark to place the sensor as close to the living tissue as possible. This ensures that hygroscopic shrinkage and swelling of dead tissues from the outer bark does not influence the diameter measurements. The sensor used had a temperature coefficient of <0.2 µm/K.<br>Additionally, we recorded on-site soil temperatures (temperature, T, °C) at a depth of 15 cm below the ground surface (within the root zone), which we assessed using ONSET's type S-TMB-002 temperature sensors (±0.2 °C accuracy). Volumetric soil water content (soil moisture, SM, m^3 water/m^3 soil), also at 15 cm below the ground surface, was measured using ONSET's type S-SMD-M005 soil moisture sensors (±3% accuracy). Data were measured at 1 min intervals and recorded as hourly means, using ONSET's HOBO loggers (type 191 H21-002). Additionally, we calculated site-specific estimates of atmospheric water demand (vapour pressure deficit, VPD, kPa) based on relative air humidity (Fig. 1e) at the ridge positions, which was obtained from Skye rht+ sensors (SKH 2065) mounted to an ADL-MX datalogger (Meier NT) and our above temperature measurements at each site, using the Magnus equation of Sonntag (1990).
Timing and spatial scale	The data was measured continiously starting from 2015/01/01 to 2020/07/31. Initially, all data was recorded hourly and then averraged (daily means) to reduce complexity and processing time for the following statistical analysis.
Data exclusions	Individual samples containing missing data were removed prior to the analysis and are thus not included in the study.
Reproducibility	This study is part of the on-going long-term alpine ecosystem research project (LTAER). With this project we tested the study design for several years, starting with the first dendrometer installations in 2012, before presenting the final study period here. Within this time period, numerous installations of technical equipment were run.
Randomization	The sampled specimens were not allocated into random groups.
Blinding	Blinding was not relevant for our study, because we studied shrub specimens.

Did the study involve field work? ☒ Yes ☐ No

Field work, collection and transport

Field conditions	The study was conducted in four alpine locations, two in Mediterranean-alpine climatic regimes in the Sierra Nevada, Spain, and two in arctic-alpine climate regimes in the Central Norwegian mountains. Each location was situated above the local treeline and characterized by a mixture of evergreen and deciduous shrub vegetation within a topographically heterogeneous landscape. Following the framework of our long-term alpine ecosystem research project (LTAER), the monitored sites at each location were placed at approximately 100-m intervals from the treeline upwards and stratified-randomly chosen to cover the elevational gradient. Within each elevational band, we monitored shrub growth and micro-site environment at distinct micro-topographic positions on

	local, exposed ridges and adjoined slopes. In accordance with the topography, these microsites experience clearly differing environmental conditions with the differences mainly caused by snow distribution, wind, and exposure to solar radiation. Thus, wind-blown ridges are characterised by discontinuous snow cover and deeply frozen ground in winter in the arctic-alpine areas. Due to the general absence of a thick layer of snow in winter, this effect was less visible at the Mediterranean ridges, which mainly distinguished themselves through slightly dryer conditions throughout the year.
Location	Study sites:<br>Norway:<br>Geiranger/Møre og Romsdal region (62°030 N, 7°150 10 E)<br>Vågåmo/Innlandet region (61°530 N, 9°150 E)<br>Spain:<br>Capilleira/Mulhacén region (37°010 N, 3°260 W)<br>Mecina region (37°050 N, 3°150 W)
Access & import/export	The access to the study sites in Norway was granted by both the landowners and Norwegian authorities (Vågå and Stranda municipalities) We also collaborated with the Geirangerfjord World Heritage Foundation and the Norwegian Fjord Centre, UNESCO World Heritage Site who permited our field work in the Geiranger World Natural Heritage and Landscape Protection Area.<br>In Spain, the access and research permission was granted by the Sierra Nevada National Park and Natural Park (project number 38_21) and the Spanish authorities of Granada Province.<br>We did not export any samples or materials from the study areas.
Disturbance	All instalations and measurements were non-invasive. No disturbance can be reported.

Reporting for specific materials, systems and methods

We require information from authors about some types of materials, experimental systems and methods used in many studies. Here, indicate whether each material, system or method listed is relevant to your study. If you are not sure if a list item applies to your research, read the appropriate section before selecting a response.

Materials & experimental systems

n/a	Involved in the study
☒	☐ Antibodies
☒	☐ Eukaryotic cell lines
☒	☐ Palaeontology and archaeology
☒	☐ Animals and other organisms
☒	☐ Human research participants
☒	☐ Clinical data
☒	☐ Dual use research of concern

Methods

n/a	Involved in the study
☒	☐ ChIP-seq
☒	☐ Flow cytometry
☒	☐ MRI-based neuroimaging
